# Analysis of Clinical Features and Risk Factors in Pregnant Women With Miliary Pulmonary Tuberculosis After *In Vitro* Fertilization Embryo Transfer

**DOI:** 10.3389/fcimb.2022.885865

**Published:** 2022-07-11

**Authors:** Siyuan Dong, Ruoyu Zhou, Emin Peng, Ruoxi He

**Affiliations:** ^1^Department of Respiratory Medicine, National Key Clinical Specialty, Branch of National Clinical Research Center for Respiratory Disease, Center of Respiratory Medicine, National Clinical Research Center for Geriatric Disorders, Xiangya Hospital, Central South University, Changsha, Hunan, China; ^2^Xiangya International Medical Center, Xiangya Hospital, Central South University, Changsha, Hunan,China

**Keywords:** miliary pulmonary tuberculosis, pregnant, infertility, *in vitro* fertilization, embryo transfer

## Abstract

**Purpose:**

Miliary pulmonary tuberculosis (TB) among pregnant women after *in vitro* fertilization embryo transfer (IVF-ET) causes poor outcomes but is rarely reported. This study analyzed the clinical characteristics and risk factors of these patients to provide hints for further studies.

**Method:**

The demographic characteristics, clinical manifestations, radiologic features, treatment, and outcomes of six patients diagnosed from May 2012 to August 2021 in Xiangya Hospital and 69 patients that were reported in English or Chinese literature from January 1980 to August 2021 were retrospectively analyzed. Continuous variables were compared between groups by *t*-test or *Mann–Whitney U* test, and categorical variables were compared between groups by *chi-square* test or *Fisher exact* test. *Univariate and multiple logistic regression* analyses were used to determine the predictors of respiratory failure.

**Results:**

A total of 75 patients were included. The average age of patients was about 30 years. All patients had tubal obstruction; 5 of them were diagnosed with pelvic TB before. Thirteen cases had a history of pulmonary or extrapulmonary TB, six out of them without any antituberculosis treatment history. All patients were in their first or second trimester during the onset of symptoms. The average interval between onset of symptoms and radiologic examination was about 21 days. The most common abnormalities on chest computed tomography scan were multiple nodules, pulmonary infiltrate, and consolidation. Merely 10 patients obtained bacteriological diagnosis by *Mycobacterium tuberculosis* culture or polymerase chain reaction test. The other patients were clinically diagnosed. All the patients received antituberculosis treatment. Although 44% of patients had fatal complications, all cases were cured or improved after antituberculosis treatment. Unfortunately, only eight fetuses survived (10.6%). The most frequent and severe complication was type I respiratory failure (20%). Patients with expectoration, dyspnea, coarse breath sounds, ground-glass opacity, and pulmonary infiltrate or consolidation were more likely to have respiratory failure (*P* < 0.05). Ground-glass opacity (OR = 48.545, 95% CI = 2.366–995.974, *P* = 0.012) and pulmonary infiltrate or consolidation (OR = 19.943, 95% CI = 2.159–184.213, *P* = 0.008) were independent predictors for respiratory failure.

**Conclusion:**

Tube infertility with underscreened or untreated TB is a risk factor for miliary TB during pregnancy after IVF-ET. Ground-glass opacity and pulmonary infiltrate or consolidation are predictors of respiratory failure. We demonstrate risk factors for incidence and complications to supply clues for future intervention and improve patient prognosis.

## Introduction

Tuberculosis (TB) is a communicable disease caused by the bacillus *Mycobacterium tuberculosis* which is a major cause of ill health and one of the leading causes of death globally. Worldwide, an estimated 9.9 million people fell ill with TB in 2020 (WHO, 2021a). Pregnancy is one of the risk factors for TB. The incidence rate ratio for TB in pregnant women is 1.4 and 1.9 times for postpartum compared with non-pregnant women (Jonsson et al., 2020). TB is a curable and preventable disease. However, the delay in the diagnosis and treatment of TB during pregnancy is associated with poor outcomes, including increased mortality in both fetuses and pregnant women (Sugarman et al., 2014). Pregnancy after *in vitro* fertilization and embryo transfer (IVF-ET) is a special and underestimated condition, which is susceptible to TB infection, especially miliary TB infection (Wang et al., 2022). However, the underlying reasons have not been clarified yet. IVF-ET is an effective technique to treat infertility with the process of *in vitro* fertilization of treated sperm and cultured mature ovum to form fertilized ovum and then implant early embryos into the uterine cavity. Genital TB (GTB) is a chronic inflammatory disease of reproductive organs involving the fallopian tubes, ovaries, pelvic peritoneum, and endometrium caused by *M. tuberculosis* with an approximately 3%–16% incidence rate in developing countries (Sharma, 2015), which is one of the most common causes of infertility (Muneer et al., 2019). Undetected and untreated GTB or other latent TB infections before IVF-ET is probably the main cause of miliary TB because the reactivation of a latent TB focus could cause miliary TB *via* hematogenous spread (Gai et al., 2021). After establishing a primary focus of infection in the lung, miliary TB affects multiple organs and systems, such as the liver, spleen, bone marrow, and brain. Miliary TB is associated with poor prognosis, which may induce not only fetal death but also life-threatening complications to patients such as respiratory failure and acute respiratory distress syndrome (ARDS) (Ma et al., 2021). Several case reports and case series referring to miliary TB after IVF-ET have been reported previously (Jacquemyn et al., 2012; Li and Zhao, 2015; Ye et al., 2019; Gai et al., 2021). Retrospective studies are rare. China is a developing country with high incidence as well as considerable burden of pregnancy TB (Sugarman et al., 2014). Most of the cases were published in Chinese by Chinese scholars without being included in previous analyses. This is a retrospective study of 69 cases that were previously reported both in the English and Chinese literatures combined with six cases diagnosed in Xiangya Hospital. The purpose of this study is to highlight the characteristics of pregnant cases with miliary pulmonary TB after IVF-ET and the probable risk factors.

## Materials and Methods

### Research Subjects in Our Hospital

This study was performed at Xiangya Hospital, Central South University (China), a 3,500-bed tertiary-care center. Six pregnant patients with miliary pulmonary TB after IVF-ET from May 2012 to August 2021 were included through a systemic search of the database in our hospital. Demographic characteristics; past medical history; clinical presentations; radiologic, laboratory, and bronchoscopic findings; diagnostic approaches; treatments; and outcomes were retrospectively extracted from medical records using a standardized protocol. The studies involving human participants were approved by the Ethics Committee of Xiangya Hospital, Central South University (No. 201906766).

### Literature Review and Data Acquisition

We conducted a MEDLINE (National Library of Medicine, Bethesda, Maryland) search with the MeSH terms (“Fertilization *in vitro*” or “embryo transfer” or “pregnancy”) and “tuberculosis” to identify literature published between January 1980 and August 2021. We found 69 relevant literatures. After excluding irrelevant ones, a total of five relevant literatures were retrieved (Addis et al., 1988; Gull et al., 1995; Jacquemyn et al., 2012; Li and Zhao, 2015; Ye et al., 2019). The literature types were case reports and retrospective studies including 10 cases with valid variables. The same retrieval strategy was adopted to search in four Chinese databases, namely, China National Knowledge Infrastructure (CNKI), Chinese Biomedical Literature Database (CBM), VIP Database for Chinese Technical Periodicals, and Wanfang Database. In total, we found 168 relevant literatures. After excluding the literatures irrelevant to the subjects as well as those that lack clear diagnostic information and outcome, 23 relevant literatures were included with a total of 59 valid cases. All data shown here were extracted from these case reports and case series; some articles occasionally lacked relevant clinical data or treatment.

### Definitions

The bacteriologically confirmed pulmonary TB is established by isolation of *M. tuberculosis* from a bodily secretion or fluid (e.g., culture of sputum, bronchoalveolar lavage fluid (BALF), or pleural fluid) or tissue (e.g., pleural biopsy or lung biopsy) (Madhukar et al., 2016). A positive nucleic acid amplification test (NAAT) amplification of the genetic material uses the polymerase chain reaction (PCR) method in a person at risk for TB (who has no prior history of treatment for TB) who is considered sufficient for diagnosis of TB (WHO, 2021b). Clinically diagnosed pulmonary TB is based on symptoms, abnormalities on chest radiography/computed tomography (CT), suggestive histology, or the clinical and radiographic improvement after antituberculosis treatment (Deng et al., 2012; WHO, 2021b). The term miliary pulmonary TB was originally a pathologic and then a radiographic description. Patients are diagnosed with miliary pulmonary TB if they have a diagnosis of TB with the hallmark radiological appearance of the involved lung covered with firm small white nodules like numerous millet seeds.

### Statistical Methods

The data were shown as mean ± standard deviation (SD) for quantitative variables and as absolute and relative frequencies (%) for qualitative variables. Continuous variables were compared between groups by *t*-test or *Mann–Whitney U* test, and categorical variables were compared between groups by *chi-square* or *Fisher exact* test. The independent risk predictors of respiratory failure were determined by *univariate and multivariate binary logistic regression* analyses. All significance tests were two-tailed tests. Statistical significance was set at *P* < 0.05. SPSS 22.0 software was used for statistical analysis.

## Results

The characteristics of six patients in our hospital are presented in **Table 1**. The average age was 30 years. The intervals from embryo transfer to the onset of symptoms were from 42 to 109 days. All six patients were diagnosed with fallopian tube obstruction before IVF-ET, and none of them underwent laparoscopy before. Two out of six patients had a history of TB infection; one of them did not accept treatment for pulmonary TB. Fever and dyspnea were complained by all the patients, which were the most common symptoms followed by cough. All patients had coarse breath sounds and moist rales on physical examination. Multiple nodules were presented among six patients, and half of the patients were illustrated with ground-glass opacity (GGO), pulmonary infiltrate or consolidation, and pleural effusion. All the patients had an elevated CRP level and neutrophil level in peripheral blood cell tests. The cultures of *M. tuberculosis* and PPD skin reaction were negative; however, T-cell enzyme-linked immunospot (T-SPOT) was positive. Patients were clinically diagnosed with pulmonary TB. Four out of six patients underwent bronchoscopy, but the changes were non-specific. Additionally, merely one patient was proved to be positive in acid-fast bacillus (AFB) smear of BALF. The primary diagnosis of patients was pneumonia, and patients were treated with broad-spectrum antibiotics as the initial treatment. Three out of them underwent respiratory failure and/or combined with ARDS. After being diagnosed, six patients received first-line antituberculosis treatments as recommended by the World Health Organization and were cured eventually. However, only one baby survived.

**Table 1:** Characteristics of six patients diagnosed with miliary pulmonary tuberculosis after IVF-ET in Xiangya Hospital.

Variables*^a^*	No. of patients
	N (%)/(mean ± SD)
Age, years [6]	30.33 ± 1.21 (29–32)
Time from received IVF-ET to onset of symptoms, days [6]	81.83 ± 25.56 (42–109)
Diagnosed with fallopian tube obstruction before IVF-ET [6]	6 (100)
Diagnosed with tuberculosis before and antituberculosis drugs treatment [6]	
Untreated pulmonary tuberculosis	1 (16.7)
Treated tuberculosis peritonitis	1 (16.7)
Clinical manifestations at diagnosis[6]*^b^*	
Fever	6 (100)
High-grade fever	3 (50)
Moderate fever	3 (50)
Dyspnea	6 (100)
Cough	5 (83.3)
Productive cough	3 (50)
Non-productive cough	2 (33.3)
Decreased appetite	4 (66.7)
Vaginal bleeding	3 (50)
Headache	2 (33.3)
Fatigue	2 (33.3)
Night sweat	1 (16.7)
Disorders of consciousness	1 (16.7)
Weight loss	1 (16.7)
Physical examination findings [6]^b^	
Coarse breath sounds	6 (100)
Moist rales	6 (100)
Radiologic examination methods [6]	
X-ray+ CT	5 (83.3)
Only CT	1 (16.7)
Interval between onset of symptoms to radiologic examination, days [6]	21.17 ± 6.91 (10–30)
Radiologic findings [6]*^b^*	
Multiple nodules	6 (100)
Ground-glass opacity	3 (50)
Pulmonary infiltrate or consolidation	3 (50)
Pleural effusion	3 (50)
Calcification	1 (16.7)
Laboratory examination	
Elevated CRP [4]	4 (100)
CRP (mg/L)	89.75 ± 38.18 (55–132)
Elevated ESR [6]	4 (66.7)
ESR (mm/h)	53.17 ± 35.41 (6–83)
Elevated neutrophils in peripheral blood cells blood tests[6]	6 (100)
PPD skin reaction positive [2]	0
T-spot positive[5]	5 (100)
Acid-fast bacilli smear positive	
Sputum [6]	1 (16.7)
BALF[4]	1 (25)
* Mycobacterium tuberculosis* culture positive	
Sputum [4]	0
BALF [3]	0
Diagnosed method [6]	
Clinically diagnosed with pulmonary tuberculosis	6 (100)
Pathological diagnosis	1 (16.7)
Bronchoscopic descriptions[6]	4 (66.7)
Inflammation	2 (33.3)
Purulent secretion	1 (16.7)
Hyperemic mucosa	1 (16.7)
Primary diagnosis [6]	
Pneumonia	6 (100)
Type of initial pharmacological therapies [6]	
Broad-spectrum antibiotics	6 (100)
Type of antituberculosis treatments [6]	
Isoniazid + rifampicin + pyrazinamide + ethambutol	5 (83.3)
Isoniazid + rifampicin + pyrazinamide	1 (16.7)
Outcomes[6]	
Cured	6 (100)
Fetal condition[6]	
Spontaneous abortion	2 (33.3)
Artificial termination of pregnancy	1 (16.7)
Stillborn and curettage	1 (16.7)
Preterm delivery and death	1 (16.7)
Survivor	1 (16.7)
Complication [6]*^b^*	
Type I respiratory failure	3 (50)
Tuberculosis meningitis	1 (16.7)
Acute respiratory distress syndrome	2 (33.3)

^a^Values in brackets represent number of patients for whom data were available.

^b^Total number of patients may be less than the sum of clinical manifestations, physical examination findings, radiologic findings, and complications, because in some cases >1 variable was present in the same patient.

IVF-ET, in vitro fertilization embryo transfer; CRP, C-reactive protein; ERS, erythrocyte sedimentation rate; BALF, bronchoalveolar lavage fluid; PCR, polymerase chain reaction.

As **Table 2** shows, all the 75 patients were of childbearing age. The mean age of patients ranged from 21 to 39 years. The intervals between embryo transfer and the onset of symptoms varied from 28 to 240 days. All patients were in their first or second trimester when symptoms appeared. In available data, 53 patients were diagnosed with unilateral or bilateral fallopian tubal obstruction. Among them, five cases had been diagnosed with pelvic TB before they received IVF-ET, two out of them diagnosed by laparoscopy. Other patients with fallopian tubal obstruction did not exclude reproductive system TB by further examinations. There were 14.7% of patients with a history of diagnosed pulmonary TB or radiologic changes inferring to latent pulmonary TB; 10.7% of patients had extrapulmonary TB including pelvic TB, tuberculosis pleurisy, or tuberculosis peritonitis. Less than a third of patients with TB and one out of five patients with pelvic TB had been treated with antituberculosis drugs before.

**Table 2:** Demographic characteristics and past medical history in 75 patients diagnosed with miliary pulmonary tuberculosis after IVF-ET.

Variables*^a^*	No. of patients
	N (%)/(mean ± SD)
Age, years [75]	30.20 ± 3.54 (21–39)
Time from IVF-ET to onset of symptoms, days [63]	82.79 ± 40.86 (28–240)
Diagnosed with fallopian tube obstruction before IVF-ET [53]	53 (100)
Type of embryos for IVF-ET [18]	
Fresh embryos	14 (77.8)
Frozen-thawed embryos	4 (22.2)
Diagnosed with tuberculosis before [75]	
None	56 (74.7)
Extrapulmonary tuberculosis	8 (10.7)
Pelvic tuberculosis	5 (6.7)
Tuberculosis pleurisy	2 (2.7)
Tuberculosis peritonitis	1 (1.3)
Latent pulmonary tuberculosis diagnosed by X-ray/CT scan	6 (8)
Pulmonary tuberculosis	5 (6.7)
Treated with antituberculosis drugs [19]	
Extrapulmonary tuberculosis [8]	
Treated	5 (29.4)
Untreated	2 (11.8)
Latent pulmonary tuberculosis [6]	
Untreated	6 (35.3)
Pulmonary tuberculosis [5]	
Treated	2 (11.8)
Untreated	2 (11.8)

^a^Values in brackets represent number of patients for whom data were available.

IVF-ET, in vitro fertilization embryo transfer.

As described in **Table 3**, the most common symptoms observed were fever (mainly high fever, ranging from 37.5°C to 40.0°C) (96.0%), cough (64.0%), dyspnea (46.7%), and vaginal bleeding (34.7%). Other symptoms suggestive of TB were uncommon, including night sweats (18.7%), decreased appetite (10.7%), fatigue (6.7%), and weight loss (4%). Besides fever, we found symptoms indicating intracranial TB infection, such as headache (18.7%) and disorders of consciousness (6.7%). Twenty-six of the 43 patients (60.5%) had positive physical findings. However, most of them were non-specific except neck stiffness (7%). A radiologic examination was done in all patients. The average interval time between the onset of symptoms and the first radiologic examination was about 21 days (range from 9 to 51 days). A percentage of 65.7% of the cases preferred to have X-ray examination other than CT scan initially; 12 of those patients (27.3%) were found to have miliary lesions in X-ray without further radiological examination. Thirty-two of 44 patients (72.7%) further underwent a CT scan for a certain diagnosis. Abnormal radiologic findings included multiple nodules (100%), pulmonary infiltrate or consolidation (32.7%), calcification (29.1%), pleural effusion (12.7%), GGO (9.1%), and fibrotic shadows (2.7%).

**Table 3:** Clinical manifestations and radiologic findings.

Variables*^a^*	No. of patients
	N (%)/ (mean ± SD)
Clinical manifestations at diagnosis[75]*^b^*	
Fever	72 (96)
High-grade fever	39 (67.2)
Moderate fever	15 (25.9)
Low-grade fever	4 (6.9)
Cough	48 (64)
Productive cough	27 (56.3)
Nonproductive cough	21 (43.7)
Dyspnea	35 (46.7)
Vaginal bleeding	26 (34.7)
Night sweat	14 (18.7)
Headache	14 (18.7)
Shiver	10 (13.3)
Decreased appetite	8 (10.7)
Fatigue	5 (6.7)
Disorders of consciousness	5 (6.7)
Weight loss	3 (4)
Physical examination findings[43]*^b^*	
Coarse breath sounds	18 (41.9)
Moist rales	18 (41.9)
Peripheral edema	4 (9.3)
Low pitched breath sounds	3 (7)
Neck stiffness	3 (7)
Radiologic examination methods[67]	
X-ray+ CT	32 (47.8)
Only CT	23 (34.3)
Only X-ray	12 (17.9)
Interval between onset of symptoms to radiologic examination, days [67]	21.46 ± 10.81 (9-51)
Radiologic findings[55]*^b^*	
Multiple nodules	55 (100)
Pulmonary infiltrate or consolidation	18 (32.7)
Calcification	16 (29.1)
Pleural effusion	7 (12.7)
Ground-glass opacity	5 (9.1)
Fibrotic shadows	2 (2.7)

^a^Values in brackets represent number of patients for whom data were available.

^b^Total number of patients may be less than the sum of clinical manifestations, physical examination findings, and radiologic findings, because in some cases >1 variable was present in the same patient.

The laboratory and bronchoscopic findings are described in **Table 4**. In the aspect of inflammation examinations, 34 of 62 patients (54.8%) had normal blood tests, and 45.2% had elevated white blood cell (WBC) count and/or increased neutrophil percentage. The erythrocyte sedimentation rate (ESR) and C-reactive protein (CRP) level were observed to be elevated in most of the patients with available variables, 75% and 100% respectively. However, the two values had large variations. In the other aspect of examinations for TB, the most common test, PPD skin reaction test (24%), had a lower positive rate than T-spot assay (95.2%). Additionally, the positive ratios of *M. tuberculosis* culture and AFB smear were also very low, 24.1% in sputum and 28.6% in BALF for *M. tuberculosis* culture, and 33.3% in sputum and 40% in BALF for AFB smear. There were also some unexpected findings in unusual species like urine, cerebrospinal fluid, and fetal chorionic. Ten patients in this study were microbiologically diagnosed with miliary pulmonary TB by *M. tuberculosis* culture (9.3%) or PCR assay (4%) without drug susceptibility testing. The rest of the patients (65 out of 75) were clinically diagnosed, four of whom had pathologic histology evidence with caseous necrosis in granulomas with/without positive AFB smear. Seven patients had the description of bronchoscopy examination (9.3%). Inflammation of the bronchus was the most common pathological change (57.1%) followed by purulent secretion (14.3%) and hyperemic mucosa (14.3%). One of them had a normal description under bronchoscopy.

**Table 4:** Laboratory examinations and bronchoscopic descriptions.

Variables*^a^*	No. of patients
	N (%)/(mean ± SD)
Laboratory examinations	
Elevated CRP[29]	29 (100)
CRP (mg/L)	55.9 ± 35.6 (11.7–132.0)
Elevated ESR [44]	33 (75)
ESR (mm/h)	47 ± 29 (6–132)
Elevated neutrophils in peripheral blood cells blood tests[62]	28 (45.2)
PPD skin reaction positive[25]	6 (24)
T-spot positive[21]	20 (95.2)
Acid-fast bacilli smear positive	
Sputum[29]	7 (24.1)
BALF [7]	2 (28.6)
Urine[1]	1 (100)
*Mycobacterium tuberculosis* culture positive	
Sputum[16]	6 (37.5)
BALF[5]	2 (40)
Cerebrospinal fluid[1]	1 (100)
Fetal chorionic[1]	1 (100)
Urine[1]	1 (100)
Blood[1]	1 (100)
Antituberculosis drug susceptibility testing	0
*Mycobacterium tuberculosis* PCR positive	
Sputum[7]	2 (28.6)
BALF[1]	1 (100)
Fetal chorionic[1]	1 (100)
Diagnosed method[75]	
Microbiological diagnosis	7 (9.3)
Sputum culture	5 (6.7)
BALF culture	2 (2.7)
PCR test	3 (4.0)
Sputum	2 (2.7)
BALF	1 (1.3)
Clinically diagnosed with pulmonary tuberculosis	65 (86.7)
Pathological diagnosis	4 (5.3)
Bronchoscopic descriptions[7]	
Inflammation	4 (57.1)
Purulent secretion	1 (14.3)
Hyperemic mucosa	1 (14.3)
Normal	1 (14.3)

^a^Values in brackets represent number of patients for whom data were available.

CRP, C-reactive protein; ERS, erythrocyte sedimentation rate; BALF, bronchoalveolar lavage fluid; PCR, polymerase chain reaction.

Initially, all the patients were misdiagnosed with pneumonia and treated with broad-spectrum antibiotics (**Table 5**). The patients subsequently received antituberculosis therapy after being microbiologically or clinically diagnosed with pulmonary TB. Antituberculosis treatment regimens were slightly different from case to case. Overall, isoniazid (H), rifampicin (R), pyrazinamide (Z), and ethambutol (E) were the most frequently used first-line antituberculosis therapy (49.3%). After the treatment, all the patients had improvement or were cured. However, merely eight of 75 fetuses (10.6%) survived. The leading reason for fetal mortality was spontaneous abortion (46.7%), followed by artificial termination of pregnancy (32%), stillbirth (7.5%), and preterm delivery and death (4%). Twenty-five percent of patients had fatal complications including respiratory failure, ARDS, and shock. The most frequent and severe complication was type I respiratory failure (15 in 75 cases, 20%); the second was tuberculosis meningitis (13 cases, 17.3%), followed by ARDS (3 cases, 4%) (**Table 4**).

**Table 5:** Treatment, outcomes, and complications.

Variables*^a^*	No. of patients
	N (%)/(mean ± SD)
Primary diagnosis[58]	
Pneumonia	58 (100)
Type of initial pharmacological therapies[58]	
Broad-spectrum antibiotics	58 (100)
Type of anti-tuberculosis treatments[75]	
Isoniazid+rifampicin+pyrazinamide+ethambutol	37 (49.3)
Isoniazid+rifampicin+pyrazinamide	4 (5.3)
Isoniazid+pyrazinamide+ethambutol+p-aminosalicylic acid	1 (1.3)
Isoniazid+ethambutol+streptomycin	1 (1.3)
Isoniazid+ethambutol	1 (1.3)
Outcomes[75]	
Improved	48 (64)
Cured	27 (36)
Fetal condition[75]	
Spontaneous abortion	35 (46.7)
Artificial termination of pregnancy	24 (32)
Survived	8 (10.6)
Stillborn and curettage	5 (6.7)
Preterm delivery and death	3 (4)
Complication[75]*^b^*	
Type I respiratory failure	15 (20)
Tuberculous meningitis and/or encephalitis	14 (18.7)
Acute respiratory distress syndrome	3 (4)
Endometrial tuberculosis	2 (2.7)
Anemia	1 (1.3)
Hypoproteinemia	1 (1.3)
Shock	1 (1.3)

^a^Values in brackets represent number of patients for whom data were available.

^b^Total number of patients may be less than the sum of complications, because in some cases >1 variable was present in the same patient.

**Figure 1:**
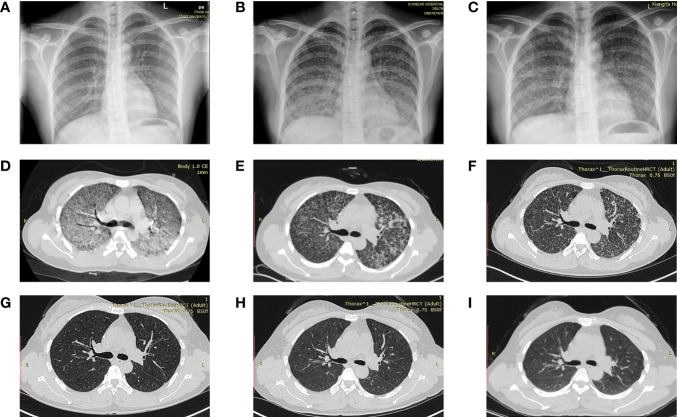
Dynamic imaging changes of a 30-year-old pregnant woman diagnosed with miliary pulmonary tuberculosis after *in vitro* fertilization and embryo transfer **(A)** Normal chest X-ray in the routine exam before IVF-ET. **(B)** A massive and symmetrical ground-glass opacity in bilateral lungs 40 days after IVF-ET. **(C)** Decreased ground-glass opacity after antituberculosis treatment. **(D)** Diffuse ground-glass opacity and partial fusion, multiple nodules, and a small amount of pleural effusion in the right thorax and a calcification nodule in the right middle lobe. **(E, F)** Resolution of miliary nodules after 1 and 2 months of antituberculosis treatment, respectively. **(G–I)** Nodules disappeared at 8 months and 1 and 3 years after treatment. IVF-ET, *in vitro* fertilization and embryo transfer.

The dynamic imaging changes of a 30-year-old pregnant woman diagnosed with miliary pulmonary TB after IVF-ET at Xiangya Hospital are shown in **Figure 1**. The routine chest X-ray before the IVF-ET procedure was normal (**A**). Half a month after IVF-ET, the patient started to have fever and shortness of breath. X-ray showed a massive and symmetrical GGO in bilateral lungs 40 days after IVF-ET (**Figure 1B**). After antituberculosis treatment for a week, imaging showed a decrease in GGO (**C**). CT scan revealed diffuse GGO with partial fusion, multiple nodules, and a small amount of pleural effusion in the right thorax and a calcification nodule in the right middle lobe (**Figure 1D**). (**Figures 1E, F**) Resolution of miliary nodules was observed after 1 and 2 months of antituberculosis treatment, respectively. (**Figures 1G–I**) CT scan at 8 months and 1 and 3 years after therapy showed that nodules disappeared in the lungs.

**Figure 2:**
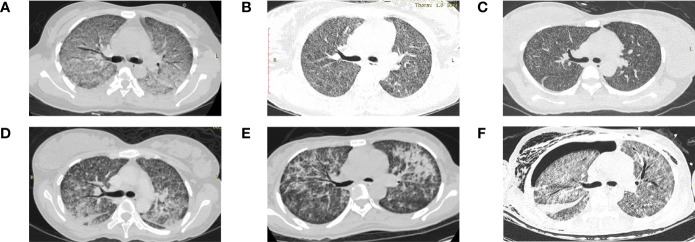
Chest computed tomography scan of six patients from Xiangya Hospital diagnosed with miliary pulmonary tuberculosis after *in vitro* fertilization and embryo transfer **(A)** Extensive ground-glass opacity in both lungs. **(B)** Indiscernible multiple nodules against the background of ground-glass shadows in both lungs. **(C)** Diffuse random multiple nodules in bilateral lungs. **(D)** Symmetric distribution of pulmonary infiltrate and consolidation in both lungs and bilateral pleural effusion. **(E)** Multiple patchy high-density shadows in both lungs. **(F)** Ground-glass opacity, pulmonary infiltrate and consolidation in both lungs, and a fluid pneumothorax at the right thorax.

Chest radiological images of six patients from Xiangya Hospital are presented in **Figure 2**. All images of six patients showed miliary and multiple nodules in the bilateral lungs. **Figure 2A** illustrates the extensive GGO. Multiple nodules were indiscernible against the background of ground-glass shadows in both lungs (**Figure 2B**). **Figure 2C** depicts diffuse random multiple nodules, which is a typical presentation of miliary pulmonary TB. **Figures 2D, E** show the symmetric distribution of pulmonary infiltrate and consolidation, accompanied by bilateral pleural effusion as shown in **Figure 2D**. GGO, pulmonary infiltrate, and consolidation were as presented in **Figure 2F**, and a fluid pneumothorax was found at the right thorax after mechanical ventilation.

The *chi-square* test showed that patients with expectoration, dyspnea, coarse breath sounds, GGO, and pulmonary infiltrate or consolidation were more likely to have respiratory failure (*P* < 0.05) (**Table 6**). Within 21 days of symptom onset, GGO was more possible to be detected by imaging (*P* < 0.05) (data not shown). *Logistic regression* analysis identified that pulmonary infiltrate and consolidation (odds ratio (OR) = 19.943, 95% confidence interval [CI] = 2.159–184.213, *P* = 0.008) and GGO (OR = 48.545, 95% CI = 2.366–995.974, *P* = 0.012) were independent predictors of respiratory failure (**Table 7**).

**Table 6:** Differences between patients with respiratory failure and non-respiratory failure.

Variables (N = 75)	Respiratory failure (N = 15)	Non-respiratory failure (N = 60)	*χ2*	*P* value
Expectoration	9/15 (60.0)	18/60 (30.0)	4.688	0.030
Dyspnea	15/15 (100)	20/60 (33.3)	21.429	<0.001
Ground-glass opacity	4/10 (40.0)	1/45 (2.2)	14.129	<0.001
Pulmonary infiltrate or consolidation	8/10 (80.0)	10/45 (22.2)	12.406	<0.001

**Table 7:** Univariate and multivariate binary logistic regression analysis showing independent radiologic predictors of respiratory failure in pregnant patients.

Variables (N = 75)	Univariate OR (95% CI)	*P* value	Multivariate OR (95% CI)	*P* value
Pulmonary infiltrate or consolidation	14.000 (2.554–76.744)	0.002	19.943 (2.159–184.213)	0.008
Calcification	1.833 (0.440–7.640)	0.405	–	–
Pleural effusion	4.393 (0.804–23.999)	0.088	–	–
Ground Ground-glass opacity	29.333 (2.793–308.027)	0.005	48.545 (2.366–995.974)	0.012

The characteristics of patients with successful pregnancies are presented in **Table 8** (Hou et al., 2005; Chu et al., 2011; Zhang, 2013; Liu et al., 2016; Wen et al., 2016; Zhang et al., 2017). When they were diagnosed with TB, three out of eight patients were in their first trimester, three patients were in the second trimester, and one patient was in the last trimester of pregnancy. Five patients had severe complications; three of them had tuberculosis meningitis, and two of them had respiratory failure. The antituberculosis treatment started immediately after diagnosis. Most patients received first-line antituberculosis therapy; only one patient was treated with p-aminosalicylic acid, one of second-line drugs. Four patients with complications were recorded using glucocorticoid accompanied by antituberculosis therapy. Merely three fetuses survived and were delivered by full-term cesarean section. The rest of the fetuses were delivered preterm, and one of them suffered from severe congenital TB. In accordance with our statistical analysis (data not shown), no significant difference was found between the patients with successful pregnancies and pregnancy termination.

**Table 8:** Characteristics of eight patients with successful pregnancies.

Case	Age, years	Time from received IVF-ET to onset of symptoms	History of tuberculosis	Clinical manifestations	Time from received IVF-ET to diagnosis of TB	Diagnosed method of pulmonary TB	Treatments	Complication	Fetal condition	References
Case 1	21	7 months	NA	Fever, cough, sputum, dyspnea, headache, disorders of consciousness	8 months	Clinically diagnosed	Isoniazid + rifampicin + pyrazinamide, hydrocortisone	Tuberculosis meningitis	Health baby, cesarean section at over 9 months	Liu et al., 2016
Case 2	31	69 days	None	Fever, dyspnea	90 days	Sputum PCR test positive	Isoniazid + rifampicin + pyrazinamide, prednisone	Type I respiratory failure	Health baby, cesarean section at term	Zhang et al., 2017
Case 3	29	70 days	None	Fever, headache	110 days	Clinically diagnosed	Isoniazid + rifampicin + pyrazinamide, prednisone	Tuberculosis meningitis	Health baby, preterm delivery at 32 weeks	Zhang et al., 2017
Case 4	34	3 months	None	Fever, cough, sputum, dyspnea, headache,	4 months	Clinically diagnosed	Isoniazid + rifampicin + pyrazinamide + ethambutol, dexamethasone	Tuberculosis meningitis, anemia, Hypoproteinemia	Health baby, preterm delivery at 7 months	Zhang, 2013
Case 5	21	13 weeks	NA	Cough, night sweat	NA	Sputum *Mycobacterium tuberculosis* culture positive	Isoniazid + pyrazinamide + ethambutol + p-Aminosalicylic acid	None	Baby diagnosed with severe congenital tuberculosis, cesarean section at term	Wen et al., 2016
Case 6	31	43 days	NA	Vaginal bleeding, dry cough, fever	60 days	Clinically diagnosed	Isoniazid + rifampicin + ethambutol,	None	Health baby, preterm delivery at over 7 months	Hou et al., 2005
Case 7	31	60 days	None	Vaginal bleeding, dry cough, fever	70 days	Clinically diagnosed	Isoniazid + rifampicin + pyrazinamide + ethambutol,	Type I respiratory failure	Health baby, preterm delivery at over 8 months	Chu et al., 2011
Case 8	29	109 days	None	Fever, cough, sputum, dyspnea	140 days	Clinically diagnosed	Isoniazid + rifampicin + pyrazinamide + ethambutol,	None	Health baby, cesarean section at term	**-**

## Discussion

Miliary TB is a potentially fatal form of TB. Approximately 15%–30% of patients with pulmonary TB during pregnancy exhibit hematogenous dissemination and suffer from miliary TB (Sobhy et al., 2017). It has been reported that the reasons for this phenomenon are associated with immune dysregulation (Mor and Cardenas, 2010), increased vascular permeability (Mali and Meena, 2018), or elevated blood lipid levels (Miele et al., 2020) during pregnancy, while miliary TB in pregnancy after IVF-ET is rare and the incidence has not been estimated. Our results reinforced previous findings that women during pregnancy after IVF-ET were more prone to miliary TB (Ye et al., 2019; Gai et al., 2021). Besides the waning of cellular immunity, latent TB infection and IVF-ET interventions were related to susceptibility to miliary TB (Singh and Perfect, 2007).

GTB is associated with 0.2% to 21% of infertility cases, mostly among women in resource-limited settings (Aliyu et al., 2004). Dam et al. detected 81 patients with unexplained infertility and repeated failure of *in vitro* fertilization in India by taking endometrial tissue or menstrual blood for *M. tuberculosis* PCR and found that 63 of them were positive (Dam et al., 2006). GTB is one of the most frequent etiologies of tubal infertility. Tubal infertility patients always present with failure to achieve a successful pregnancy after 12 months or more of regular unprotected intercourse in a woman combined with a history of tubal ligation or tubal changes including occlusion, hydrosalpinx, beading by hysterosalpingography, or laparoscopy (Briceag et al., 2015). *M. tuberculosis* can lead to fallopian tube inflammation, resulting in fallopian tube swelling, ponding, and intimal hyperplasia. In our study, most of the patients diagnosed with fallopian tube occlusion did not undergo laparoscopy or further examinations to exclude GTB. If IVF-ET is performed in GTB patients, *M. tuberculosis* in the original extrapulmonary lesions will spread to multiple systems through blood and lymph, resulting in miliary TB and fetal infection, even death (Ghosh et al., 2011). Extrapulmonary TB infections including GTB usually occur *via* hematogenous spread from the lungs (Aliyu et al., 2004; Moule and Cirillo, 2020). Pulmonary TB lesions such as calcification, fibrotic shadows, and pleural thickening were detected in routinely chest X-ray examination before IVF-ET in some cases here. The activation of latent *M. tuberculosis* may be the main reason for miliary TB in pregnant patients after IVF-ET.

As far as I know, there is lack of international guidelines and consensus on the TB screening procedures in patients with tubal occlusion before IVF-ET. Nowadays, tuberculosis is not uncommon in developed countries because of the increasing number of immigrants. However, TB screening is not part of the routine tests in infertility patients in developed countries (Jacquemyn et al., 2012). Laparoscopy was executed in all of three patients reported before in developed countries (Addis et al., 1988; Gull et al., 1995; Jacquemyn et al., 2012). It seems that laparoscopy is a usual examination not for TB screening but for infertility patients. In China, due to the high prevalence and burden of TB, TB screening by X-ray is the routine examination before IVF-ET nowadays. On the contrary, because of the limited medical and financial resources, only patients with risk factors for active TB infection will be asked to complete laparoscopy, including relevant clinical manifestations (pelvic or abdominal pain, and/or menstrual disorders) or classical changes under hysterosalpingography (fallopian tube constriction and/or uterine cavity adhesion or deformity, especially beaded tubal), as well as relevant epidemiologic factors (history of prior TB infection without antituberculosis treatment) and/or radiographic findings referring to active TB infection (Malhotra et al., 2020).

Another possible cause of miliary TB after IVF-ET is the interventions in IVF-ET. Artificial insemination requires progesterone to support luteal function and promote embryonic development. At the same time, estrogen, progesterone, and human chorionic gonadotropin *in vivo* are significantly higher than physiological levels. These hormones have a direct inhibitory effect on CD4^+^ T lymphocytes and change the ratio of helper T lymphocyte cells (Th cells) to regulatory T cells (Treg cells) (Schumacher, 2017). CD4^+^ T lymphocytes play an important role in the infection of *M. tuberculosis*. Th cells help to enhance immune function, while Treg cells can inhibit immune response (Ghosh et al., 2011). The imbalance of the proportion of these two cells will be conducive to the spread of *M. tuberculosis*. On the other hand, adrenocortical hormones will be used to improve endometrial receptivity in some conditions, which could inhibit the organic immune system as well (Plaks et al., 2006). Therefore, the changes in hormone levels in pregnant women during pregnancy easily lead to the new infection of TB, or the reactivation and diffusion of latent *M. tuberculosis*.

In general, it is recommended that patients with high prevalence and burden of TB regions should have more tests before assisted reproduction except X-ray to exclude latent TB infection and avoid TB dissemination. Tuberculin skin test (TST) is probably the most cost-effective test for latent TB screening. The combination of TST, interferon-gamma release assay (IGAR), or molecular WHO-recommended rapid diagnostic tests (WHO, 2021c), if available, is recommended among patients with latent lesions of TB on chest radiography as well. Tubal infertility patients with the abovementioned high-risk factors are still recommended to execute laparoscopy before IVF-ET. The guidelines or protocols for screening before IVF-ET should be formulated in the future.

The early clinical manifestations of miliary pulmonary TB are diverse and non-specific (Sharma and Mohan, 2017). The most common symptoms in this study were high-level fever, cough, and dyspnea which were consistent with studies before (Ye et al., 2019; Gai et al., 2021). The most common symptom was fever because miliary TB causes fulminant infection and systemic inflammatory response. Half or more cases had increased neutrophils in blood test, ESR, and CRP in this study. T-SPOT is one kind of IGRAs, which is not affected by Bacille Calmette-Guérin (BCG) vaccination or most *non-tuberculous mycobacteria* (Pai et al., 2008). The sensitivity of T-SPOT appears to be higher than TST (approximately 90% vs. 80%) (Pai et al., 2008). The higher sensitivity of T-SPOT may be useful for evaluating individuals with immunosuppressive conditions. In this study, the positive rate of T-SPOT was much higher than TST; the possible reason is that patients with miliary pulmonary TB after IVF-ET are in a relative anergy condition. The etiological examination is critical because it is the gold standard for the diagnosis of TB, but it may be less feasible in some circumstances (Pai et al., 2016). Of the 4.8 million people diagnosed with pulmonary TB worldwide in 2020, 59% were bacteriologically confirmed (WHO, 2021a). At least 15% to 20% of patients with clinical diagnosis of TB have never been identified by specific bacteriology (CDC, 2015). In our study, only 10 patients had confirmed diagnosis. None of the drug susceptibility tests had been reported in the cases. The possible reason is the unavailability of drug susceptibility tests. In China, due to pregnancy, those patients had been diagnosed mostly at general hospitals rather than at specialized tuberculosis hospitals which have laboratories to perform all tests relevant to TB including drug susceptibility tests. In the United States, not all laboratories perform all tests too. All United States jurisdictions require the submission of culture isolates identified as *M. tuberculosis* complex (MTBC) by any laboratory to their jurisdictional public health laboratory for identification and drug susceptibility testing. Another possible reason is that physicians had insufficient knowledge and did not pay attention to drug-resistant TB because most of the patients did not have risk factors for drug-resistant TB. Merely three patients were diagnosed by PCR assay here. How to get the microbiological detection of TB as early as possible is the trickiest problem. The amplification and detection of MTBC nucleic acids is a technology that has proven to be highly sensitive and specific. WHO recommended that Xpert MTB/RIF should be used as an initial diagnostic test for TB in sputum rather than smear microscopy/culture. However, the use of rapid tests remains far too limited because it is expensive and unreachable in some areas. Among the 49 countries in one of WHO’s three global lists of high burden countries (for TB, HIV-associated TB and multidrug-resistant TB or rifampicin-resistant TB), only 21 countries reported that a WHO-recommended rapid diagnostic test had been used as the initial test for more than half of their notified TB cases (WHO 2021b). Seven cases in our series had reported bronchoscopic examination; six out of them had unspecific positive findings. Bronchoscopy is an option to obtain BALF or tissue specimens when the diagnosis is difficult and sputum is not available.

After establishing a focus of infection in the lung, bacilli can disseminate *via* the hematogenous to the most vascular organs, such as brain meningeal involvement, which was evident postmortem in 54% of cases of miliary TB (Mali and Meena, 2018). In our study, 14 out of 75 cases (18.7%) were complicated with TB meningitis and/or encephalitis. Two cases were diagnosed with endometrial TB after IVF-ET. Most clinical features of miliary TB have low specificity, which may lead to incorrect and delayed diagnosis of TB. All the patients were misdiagnosed with bacterial pneumonia and accepted antibodies therapy initially in our study. The probable reasons are non-specific presentation, elevated infection tests, and suspended radiological examination. This study reminds us that if a pregnant woman has high-grade fever and shortness of breath and does not respond to antibiotic treatment, pulmonary TB infection should be alerted and radiographic examination, TB screening, and even antituberculosis treatment should be started immediately. Since the sensitivity of culture and AFB smear is low, it suggests the need to utilize more sensitive assays like Xpert MTB/RIF and to obtain other sample sources as possible, such as urine, blood, and cerebrospinal fluid specimens.

Miliary pulmonary TB presents with acute onset and rapid clinical course. However, the delay of X-ray or CT examination among pregnant patients is common resulting from the fear of radiation exposure. Most of patients choose to accept chest X-ray instead of a more sensitive CT scan at first. Our study found that merely 27.3% of the 44 patients who underwent an X-ray examination showed typical miliary nodules. Previous research had shown that 50% of patients did not see typical manifestations on chest X-rays at the early onset, because it can be displayed under X-ray only in the presence of caseous material (Hunter, 2018). CT has a better distinguishability to the distribution of nodules than chest X-ray (Lee et al., 2014). All patients in this study were found to have miliary nodules in bilateral lungs by CT scan. GGO was discovered in 9.1% of patients here and 12.9% reported before (Han et al., 2009), indicating the beginning and rapid progress of miliary pulmonary TB (Im et al., 1995). A study found that patients with GGO exceeding 50% of the area of bilateral lung had a higher level of acute inflammatory indexes and were more prone to dyspnea, even respiratory failure and ARDS (Lee et al., 2014). Numerous tiny granulomas overlapped by the exudative lesions TB are not easily distinguished at the early stages of miliary pulmonary TB (McGuinness et al., 1992). We found that GGO was more likely to be found by chest imaging within 3 weeks after the onset of symptom. This imaging change needs to be differentiated from many other diseases, such as pneumocystis carinii pneumonia, acute interstitial pneumonia, alveolar protein deposition, and diffuse alveolar hemorrhage. A ground-glass shadow can be used as a CT sign indicating cases with active TB infection with high amounts of *M. tuberculosis* and a strong delayed hypersensitivity response of the body (Im et al., 1993). Our results reinforce preceding observation by showing that the presentation of GGO is not only correlated with the presence of type I respiratory failure but also an independent risk prognostic factor of respiratory failure (Hashemian et al., 2015). Apart from that, pulmonary infiltrate or consolidation formed by the merge of vast granulomatous nodules is also an independent risk factor for respiratory failure (Herreros et al., 2018). When the imaging found a large area of GGO and pulmonary infiltrate or consolidation, we should be alert to the occurrence of ARDS and respiratory failure among miliary pulmonary TB patients. The incidence of miliary pulmonary TB in combination with ARDS is low, but the mortality rate is high, up to 47.06% (Kim et al., 2008).

Although all patients in our study survived and had a good response to antituberculous treatment combined with respiratory support including mask oxygen inhalation or mechanical ventilation, there were still some patients that died due to TB dissemination (Ma et al., 2021). Perhaps, women who survived from miliary TB can no longer conceive again. TB in pregnancy is associated with adverse fetal consequences like a roughly two-fold increased risk of premature birth, low birthweight, and intrauterine growth retardation, and a six-fold increased risk of perinatal death (WHO, 2021a). Only eight fetuses survived; the others had been spontaneous or induced abortions in this study. All the patients with successful pregnancies received first-line antituberculosis therapy, and one of them was treated with p-aminosalicylic acid. No deformity was found. Although the teratogenic risk of normal doses of antituberculosis drugs is very low, it may still cause some injury to the fetus, such as ototoxicity (Miele et al., 2020). No significant risk factors for fetal prognosis were identified in this study; intrauterine hypoxia will adversely affect the embryo. Early recognition of patients at high risk of respiratory failure and ARDS in disseminated TB infection is vital to improving the prognosis. Postnatal, one baby was reported with congenital TB. Similar cases with congenital TB after IVF-ET have been reported before (Zhang et al., 2018). The TB screening before IVF-ET is essential to decreasing the prevalence of congenital TB in neonates.

As far as we know, we have the largest sample size of pregnant patients with miliary pulmonary TB after IVF-ET. The strength of this study is that the characteristics of six patients of Xiangya Hospital were summarized and confirmed by the analysis of other patients included. In particular, the large extent of GGO in the early stage was found among patients with dyspnea in our hospital which had been proved to be an independent risk factor of respiratory failure, and the notion has not been reported before. Another highlight of this study is that the features of patients with successful pregnancies were analyzed, which could apply experiences to other doctor counterparts. The present study has several limitations. Firstly, this was a single-center study; however, our hospital has cooperation with one of the largest reproductive specialty hospitals in China. Our patients come from all over the country and are representative. Secondly, the data for pooled analysis of retrospective studies are unavoidably incomplete in origin. We also acknowledge that not all the analyses could yield reliable results because of the relatively small sample size and missing data. However, pregnant patients with miliary TB after IVF-ET are rare and it is difficult to obtain a large multicenter series of cases for a prospective design. Therefore, our results should be considered as hypothesis-generating for future studies. Further studies are required to clarify whether complete screening of latent TB infection before IVF-ET could decrease the incidence of miliary TB during pregnancy and whether early awareness of the possibility of activation TB infection and accurate judgment of the severity of the condition could improve the maternal and fetal prognoses.

## Conclusion

After IVF-ET, patients with latent GTB or pulmonary TB are prone to the spread of *M. tuberculosis* resulting in miliary TB to the organs with rich blood supply including lung, brain, and the reproductive system due to the change of immune environment in pregnancy as well as the IVF-ET intervention. The coexistence of primary tube infertility and untreated pulmonary or extrapulmonary TB are risk factors for miliary TB. Screening patients with TB infection in high TB burden regions should be an important evaluation before IVF-ET. Unspecific manifestations, lack of awareness, and fear of radiation exposure could induce the delay of diagnosis and treatment of miliary pulmonary TB consequently leading to serious complications, poor prognosis, and even death. The appearance of specific radiographic findings especially GGO suggests that the patients are experiencing early stage and rapid progression of disease and are likely to suffer from respiratory failure. They need more attention and positive medication treatment and respiratory support therapy as well to improve the prognosis.

## Data Availability Statement

The original contributions presented in the study are included in the article/supplementary material. Further inquiries can be directed to the corresponding authors.

## Ethics Statement

The studies involving human participants were reviewed and approved by the Ethics Committee of Xiangya Hospital, Central South University. The patients/participants in Xiangya Hospital provided their written informed consent to participate in the study. Informed consent was obtained from the individual(s) for the publication of any potentially identifiable images or data included in this article.

## Author Contributions

SD and RZ collected the data. SD and RZ wrote the draft of the manuscript. RH and EP designed and edited the manuscript. RH conducted the study. All authors contributed to the article and approved the submitted version.

## Funding

This work was supported by the Natural Science Foundation of Hunan Province (grant numbers: 2020JJ5897, 2020JJ4904). The National Natural Science Foundation of China (grant number:81400022)

## Conflict of Interest

The authors declare that the research was conducted in the absence of any commercial or financial relationships that could be construed as a potential conflict of interest.

## Publisher’s Note

All claims expressed in this article are solely those of the authors and do not necessarily represent those of their affiliated organizations, or those of the publisher, the editors and the reviewers. Any product that may be evaluated in this article, or claim that may be made by its manufacturer, is not guaranteed or endorsed by the publisher.
